# Supplementary material for: The Emergence of Chikungunya ECSA Lineage in a Mayaro Endemic Region on the Southern Border of the Amazon Forest
Source: Trop Med Infect Dis. 2020 Jun 26;5(2):105. doi: 10.3390/tropicalmed5020105 (PMC7345197; doi:10.3390/tropicalmed5020105)
Supplement: Supplementary file 1 [file tropicalmed-05-00105-s001.zip › Supplementary table 2 - genomes used for phylogenetic analysis.docx]

**Supplementary data**

**Table S2.a**: Sixty-six complete Mayaro virus genomes, belonging to the D and L genotypes, used to reconstruct ML phylogenetic analysis.

| **Accession code** | **Isolate ID** | **Geographic location (country_state_city)** | **Collection date (year_month_day)** | **Source** |
| --- | --- | --- | --- | --- |
| AF237947 | VMAY | Brazil_Para | NA | NA |
| DQ001069 | MAYLC | French Guiana_Cayenne | 1998 | Human |
| KJ013266 | BNI_1_imported to Germany | French Guiana | 2013_08 | Human |
| KM400591 | Acre27 | Brazil_Acre_Acrelandia | 2004_06 | Human |
| KP842794 | MAYV16A | Venezuela_Portuguesa_La Estacion | 2010 | Human |
| KP842795 | MAYV11A | Venezuela_Portuguesa_La Estacion | 2010 | Human |
| KP842796 | MAYV12A | Venezuela_Portuguesa_La Estacion | 2010 | Human |
| KP842797 | MAYV13A | Venezuela_Portuguesa_La Estacion | 2010 | Human |
| KP842798 | MAYV14A | Venezuela_Portuguesa_La Estacion | 2010 | Human |
| KP842799 | MAYV15A | Venezuela_Portuguesa_La Estacion | 2010 | Human |
| KP842800 | ARV0565 | Peru_San Martin | 1995_04 | Human |
| KP842801 | IQE2777 | Peru_Loreto | 2006 | Human |
| KP842802 | BeAn343102 | Brazil_Para | 1978_05 | Monkey |
| KP842803 | BeH343148 | Brazil_Para | 1978_04 | Human |
| KP842804 | BeAn337622 | Brazil_Para | 1978 | Monkey |
| KP842805 | FSB0319 | Bolivia | 2002_04 | Human serum |
| KP842806 | FSB1131 | Bolivia | 2006 | Human |
| KP842807 | Ohio | Peru_Loreto | 1995 | Human |
| KP842808 | Iqu3056 | Peru_Loreto | 2000_06 | Human serum |
| KP842809 | BeH186258 | Brazil_Amapa | 1970_06 | Human |
| KP842811 | FMD0641 | Peru_Puerto Maldonato | 2005 | Human |
| KP842812 | FMD3213 | Peru_Puerto Maldonato | 2010 | Human |
| KP842814 | FVB0112 | Bolivia | 2006 | Human |
| KP842815 | FPI1761 | Peru_Iquitos | 2011 | Human |
| KP842817 | FVB0069 | Bolivia | 2006 | Human |
| KP842818 | BeAr505411 | Brazil_Para | 1991_03 | *Haemagogus janthinomys* |
| KP842819 | BeH256 | Brazil_Para | 1955_04 | Human serum |
| KP842820 | BeAr30853 | Brazil_Para | 1961_05 | *Ixodes* spp. |
| KT818520 | LPV01 imported from Portal Para | Brazil_SP_Sao Jose do Rio Preto | 2014_11_27 | Human |
| KX496990 | HsHaiti-1-15 | Haiti | 2015_01_08 | Human |
| KY026195 | FPI_1766 | Peru | NA | Human |
| KY026197 | FPI_1738 | Peru | NA | Human |
| KY026198 | FPY_0046 | Peru_Yurimaguas | 2011 | Human |
| KY026199 | FPY_0122 | Peru | NA | Human |
| KY026200 | FPI_179 | Peru_Iquitos | 2011 | Human |
| KY618127 | BeAr20290 | Brazil_Para | 1960 | *Haemagogus* spp. |
| KY618128 | BeAr344910 | Brazil_Para_Santarem | 1978 | *Haemagogus janthinomys* |
| KY618129 | BeAr505578 | Brazil_Para_Benevides | 1991 | *Haemagogus janthinomys* |
| KY618130 | BeAr757954 | Brazil_ Rio Grande do Sul_Eldorado do Sul | 2011 | *Culex (Cux.)* spp. |
| KY618131 | BeH342916 | Brazil_Para_Santarem | 1978 | Human |
| KY618132 | BeH394885 | Brazil_Para_Conceicao do Araguaia | 1981 | Human |
| KY618133 | BeH473130 | Brazil_Para_Santarem | 1988_05 | Human |
| KY618134 | BeH504639 | Brazil_Goias_Goiania | 1991 | Human |
| KY618135 | BeH505465 | Brazil_Para_Belem | 1991 | Human |
| KY618136 | BeH743921 | Brazil_Para_Santa Barbara do Para | 2008 | Human |
| KY618137 | BeH744141 | Brazil_Para_Belem | 2008 | Human |
| KY618138 | BeH744173 | Brazil_Para_Santa Barbara do Para | 2008 | Human |
| KY618139 | BeH758762 | Brazil_Para_Parauapebas | 2009 | Human |
| KY618140 | BeH792430 | Brazil_Para_Barcarena | 2012_05_04 | Human |
| KY985361 | HsHaiti-1-14 | Haiti_Gressier | 2014_06_04 | Human |
| MH513597 | H307 | Brazil_Mato Grosso_Sinop | 2015_03_16 | Human |
| MK070491 | IQT4235 | Peru_Loreto | 1997_08 | Human |
| MK070492 | TRVL_4675 | Trinidad_Mayaro Country | 1954_08 | Human serum |
| MK288026 | Venezuela_1 | Venezuela | 2016_05_02 | Human |
| MK573238 | BeH407 | Brazil_Para_Belem | 1955 | Human |
| MK573239 | BeH428890 | Para_Belem | 1984_12 | Human |
| MK573240 | TRVL15537 | Trinidad_Rio Grande Forest | 1957_03 | *Coquillettidia venezuelensis* |
| MK573241 | BeH506151 | Brazil_Tocantins | 1991 | Human |
| MK573242 | Obs2209 | Peru_Tumbes | 1995_03 | Human |
| MK573243 | Iqu2950 | Peru_Loreto | 2000_06 | Human serum |
| MK573244 | BeH343155 | Brazil_Para | 1978_04 | Human |
| MK573245 | FSB0311 | Bolivia | 2002_03 | Human serum |
| MK573246 | Uruma | Bolivia_Uruma | 1955_03 | Human |
| MK837006 | Haiti_0380 | Haiti | 2014_10_03 | Human |
| MK837007 | Haiti_0737 | Haiti | 2014_10_03 | Human |
| MN138459 | Haiti_0729 | Haiti | 2014_12_25 | Human |

**Table S2.b**: 930 complete and near-complete Chikungunya virus genomes, belonging to the West African, East/Central/South African and Asian/Caribbean genotypes, used to reconstruct ML phylogenies.

| **Accession code** | **Isolate ID** | **Geographic location**  **(country_state_city)** | **Collection date (year_month_day)** | **Source** |
| --- | --- | --- | --- | --- |
| AB455493 | SL11131 | NA | 2006_12 | Human |
| AB455494 | SL10571 | NA | 2006_12 | Human |
| AB860301 | CHIKV_13_112A | Philippines | 2013 | Human |
| AF369024 | S27_African_prototype | NA | NA | Unknown |
| AF490259 | Ross | Thailand | NA | Unknown |
| AM258990 | 05_115 | Reunion | NA | Unknown |
| AM258991 | 05_209 | Seychelles | NA | Unknown |
| AM258992 | 06_021 | Reunion | NA | Unknown |
| AM258993 | 06_027 | Reunion | NA | Unknown |
| AM258994 | 06_049 | Reunion | NA | Unknown |
| AM258995 | 05_061 | Reunion | NA | Unknown |
| AY726732 | 37997 | Senegal | NA | *Aedes furcifer* |
| DQ443544 | LR2006_OPY1 | Reunion | NA | Human |
| EF012359 | D570_06 | Mauritius | NA | Unknown |
| EF027134 | IND_06_AP3 | India | NA | Unknown |
| EF027135 | IND_06_KA15 | India | NA | Unknown |
| EF027136 | IND_06_MH2 | India | NA | Unknown |
| EF027137 | IND_06_RJ1 | India | NA | Unknown |
| EF027138 | IND_06_TN1 | India | NA | Unknown |
| EF027139 | IND_00_MH4 | India | NA | Unknown |
| EF027140 | IND_63_WB1 | India | NA | Unknown |
| EF027141 | IND_73_MH5 | India | NA | Unknown |
| EF210157 | DRDE_06_DRDEHydISW06 | India | 2006 | Human |
| EF452493 | AF15561 | Thailand | NA | Unknown |
| EF452494 | TSI_GSD_218_VR1 | USA | NA | Unknown |
| EU037962 | Wuerzburg | Mauritius | NA | Unknown |
| EU244823 | ITA07_RA1 | Italy | 2007 | Unknown |
| EU372006 | DRDE_07 | India | 2007_06_11 | Human |
| EU564334 | TM25 | Mauritius | 2006_02_14 | Human |
| EU564335 | CHIK31 | India | 2006_10_31 | Human |
| EU703759 | MY002IMR_06_BP | Malaysia | 2006 | Human |
| EU703760 | MY003IMR_06_BP | Malaysia | 2006 | Human |
| EU703761 | MY019IMR_06_BP | Malaysia | 2006 | Human |
| EU703762 | MY021IMR_06_BP | Malaysia | 2006 | Human |
| FJ000062 | IND_GJ52 | India | 2006_09 | Human |
| FJ000063 | IND_KA52 | India | 2006_10 | Human |
| FJ000064 | IND_GJ51 | India | 2006_09 | Human |
| FJ000065 | IND_GJ53 | India | 2006_09 | Human |
| FJ000066 | IND_KR51 | India | 2006_09 | Human |
| FJ000067 | IND_MH51 | India | 2006_08 | Human |
| FJ000068 | IND_KA51 | India | 2006_08 | Human |
| FJ000069 | IND_KR52 | India | 2007_06 | Human |
| FJ445426 | LKEHCH13908 | Sri Lanka | 2008_04 | Human |
| FJ445427 | LKMTCH2707 | Sri Lanka | 2007_07 | Human |
| FJ445428 | LKRGCH1507 | Sri Lanka | 2007_05 | Human |
| FJ445430 | SGEHICHD93508 | Singapore | 2008_07 | Human |
| FJ445431 | SGEHICHS421708 | Singapore | 2008_07 | Human |
| FJ445432 | SGEHICHS422308 | Singapore | 2008_07 | Human |
| FJ445433 | SGEHICHS422808 | Singapore | 2008_08 | Human |
| FJ445443 | SGEHICHS424108 | Singapore | 2008_08 | Human |
| FJ445445 | SGEHICHS425208 | Singapore | 2008_08 | Human |
| FJ445463 | SGEHICHD96808 | Singapore | 2008_07 | Human |
| FJ445484 | SGEHICHT077808 | Singapore | 2008_05 | Human |
| FJ445502 | SGEHICHD122508 | Singapore | 2008_08 | Human |
| FJ445510 | SGEHICHS277108 | Singapore | 2008_01 | Human |
| FJ445511 | SGEHICHD13508 | Singapore | 2008_01 | Human |
| FJ513628 | LK_PB_CH1008 | Sri Lanka | 2008_03 | Human |
| FJ513629 | LK_PB_CH1608 | Sri Lanka | 2008_03 | Human |
| FJ513632 | LK_PB_CH3008 | Sri Lanka | 2008_03 | Human |
| FJ513635 | LK_PB_CH5308 | Sri Lanka | 2008_03 | Human |
| FJ513637 | LK_PB_CH5808 | Sri Lanka | 2008_03 | Human |
| FJ513645 | LK_EH_CH4408 | Sri Lanka | 2008_04 | Human |
| FJ513654 | LK_EH_CH6708 | Sri Lanka | 2008_04 | Human |
| FJ513657 | LK_EH_CH7708 | Sri Lanka | 2008_04 | Human |
| FJ513673 | LK_EH_CH17708 | Sri Lanka | 2008_04 | Human |
| FJ513675 | LK_EH_CH18608 | Sri Lanka | 2008_04 | Human |
| FJ513679 | LK_EH_CH20108 | Sri Lanka | 2008_04 | Human |
| FJ807896 | 0611aTw | Singapore | 2006 | Human |
| FJ807897 | 0706aTw | Indonesia | 2007 | Human |
| FJ807898 | 0810aTw | Bangladesh | 2008 | Human |
| FJ807899 | 0810bTw | Malaysia | 2008 | Human |
| FJ959103 | BNI_CHIKV_899 | Mauritius | 2006 | Human |
| FN295483 | MY_06_37348 | Malaysia | 2006_03 | Human |
| FN295484 | MY_06_37350 | Malaysia | 2006_03 | Human |
| FN295485 | MY_08_065 | Malaysia | 2008 | Human |
| FN295487 | MY_08_068 | Malaysia | 2008 | Human |
| FR687340 | MY_08_9405 | Malaysia | 2008_08_21 | Human |
| FR687341 | MY_08_0978 | Malaysia | 2008_11_05 | Human |
| FR687342 | MY_08_9487 | Malaysia | 2008_11_10 | Human |
| FR687343 | MY_08_4567 | Malaysia | 2008_12_12 | Human |
| FR687344 | MY_09_4506 | Malaysia | 2009_01_12 | Human |
| FR687345 | MY_09_0873 | Malaysia | 2009_02_05 | Human |
| FR687346 | MY_09_5557 | Malaysia | 2009_02_16 | Human |
| FR687347 | MY_09_3467 | Malaysia | 2009_02_12 | Human |
| FR687348 | MY_09_3611 | Malaysia | 2009_04_03 | Human |
| FR717336 | IMTSSA6424S | France | 2005_12_26 | Human |
| FR717337 | IMTSSA6424C | France | 2005_12_26 | Human |
| FU759460 | UNKNOWN_FU759460 | NA | NA | Unknown |
| FU759461 | UNKNOWN_FU759461 | NA | NA | Unknown |
| FU759462 | UNKNOWN_FU759462 | NA | NA | Unknown |
| FU759463 | UNKNOWN_FU759463 | NA | NA | Unknown |
| FU759464 | UNKNOWN_FU759464 | NA | NA | Unknown |
| FU759465 | UNKNOWN_FU759465 | NA | NA | Unknown |
| GM839372 | UNKNOWN_GM839372 | NA | NA | Unknown |
| GM839373 | UNKNOWN_GM839373 | NA | NA | Unknown |
| GM839374 | UNKNOWN_GM839374 | NA | NA | Unknown |
| GM839375 | UNKNOWN_GM839375 | NA | NA | Unknown |
| GM839376 | UNKNOWN_GM839376 | NA | NA | Unknown |
| GM839377 | UNKNOWN_GM839377 | NA | NA | Unknown |
| GQ428210 | RGCB03_KL06 | India | 2006_10_07 | Human |
| GQ428211 | RGCB05_KL06 | India | 2006_10_07 | Human |
| GQ428212 | RGCB80_KL07 | India | 2007_07_12 | Human |
| GQ428213 | RGCB120_KL07 | India | 2007_07_13 | Human |
| GQ428214 | RGCB355_KL08 | India | 2008_06_29 | Human |
| GQ428215 | RGCB356_KL08 | India | 2008_05_29 | Human |
| GQ905863 | CU_Chik661 | Thailand | 2009_05_25 | Human |
| GU013528 | LK_PB_chik3408 | Sri Lanka | 2008_03 | Human |
| GU013529 | LK_PB_chik6008 | Sri Lanka | 2008_03 | Human |
| GU013530 | LK_EH_chik19708 | Sri Lanka | 2008_04 | Human |
| GU189061 | SL15649 | Sri Lanka | 2006 | Human |
| GU199350 | FD080008 | China | 2008 | Human |
| GU199351 | SD08Pan | China | 2008 | Human |
| GU199352 | FD080178 | China | 2008 | Human |
| GU199353 | FD080231 | China | 2008 | Human |
| GU301779 | CU_Chik009 | Thailand | 2009_09_04 | Human |
| GU301780 | CU_Chik10 | Thailand | 2008_10_21 | Human |
| GU301781 | CU_Chik683 | Thailand | 2009_07_27 | Human |
| GU908223 | CU_Chik_OBF | Thailand | 2009_08_14 | Mosquito |
| HE806461 | NC_2011_568 | New Caledonia | 2011_02_28 | Human |
| HM045784 | DakAr_B_16878 | Central African Republic | 1984_11_19 | *Anopheles (Ceilia) funestus* |
| HM045785 | PM2951 | Senegal | 1966_11 | *Aedes aegypti* |
| HM045786 | IbH35 | Nigeria | 1964_07_07 | Human |
| HM045787 | SV0444_95 | Thailand | 1995 | Human |
| HM045788 | PO731460 | India | 1973 | Human |
| HM045789 | 6441_88 | Thailand | 1988 | Human |
| HM045790 | PhH15483 | Philippines | 1985_07_17 | Human |
| HM045791 | JKT23574 | Indonesia | 1983 | Human |
| HM045792 | Vereeniging | South Africa | 1956_04 | Human |
| HM045793 | CAR256 | Central African Republic | NA | Unknown |
| HM045794 | DHS4263_Calif_AB | USA | 2006 | Human |
| HM045795 | SAH2123 | South Africa | 1976 | Human |
| HM045796 | CO392_95 | Thailand | 1995 | Human |
| HM045797 | RSU1 | Indonesia | 1985 | Human |
| HM045798 | SH2830 | Senegal | 1966_11 | Human |
| HM045799 | SL_CR_3 | Sri Lanka | 2007 | Human |
| HM045800 | Hu_85_NR_001 | Philippines | 1985 | Human |
| HM045801 | SL_CK1 | Sri Lanka | 2007 | Human |
| HM045802 | K0146_95 | Thailand | NA | Unknown |
| HM045803 | I_634029 | India | 1963_11_06 | Human |
| HM045804 | IPD_A_SH_2807 | Senegal | NA | Human |
| HM045805 | AR_18211 | South Africa | 1976 | *Aedes furcifer* |
| HM045807 | IbAn4824 | Nigeria | 1965_04_13 | Mouse |
| HM045808 | 3412_78 | Thailand | 1978 | Human |
| HM045809 | LSFS | Democratic Republic of the Congo | 1960 | Human |
| HM045810 | TH35 | Thailand | 1958 | Human |
| HM045811 | Ross_low_psg | Tanzania | 1953_02_22 | Human |
| HM045812 | UgAg4155 | Uganda | 1982 | Human |
| HM045813 | Gibbs_63_263 | India | 1963_11_06 | Human |
| HM045814 | 1455_75 | Thailand | 1975 | Human |
| HM045815 | ArD_30237 | Senegal | 1979_02 | *Aedes luteocephalus* |
| HM045816 | SH_3013 | Senegal | 1966_11_23 | Human |
| HM045817 | HD_180760 | Senegal | 2005_11 | Human |
| HM045818 | ArA_2657 | Cote dIvoire | 1981_09 | *Aedes luteocephalus* |
| HM045819 | ArD_93229 | Senegal | 1993_02 | *Aedes dalzieli* |
| HM045820 | ArA_30548 | Cote dIvoire | 1993_12 | *Aedes africanus* |
| HM045821 | A301 | Senegal | 1963_03_20 | Bat (*Chiroptera*) |
| HM045822 | HB78 | Central African Republic | 1978_10 | Human |
| HM045823 | Angola_M2022 | Angola | 1962 | Unknown |
| HQ456251 | Com25 | NA | NA | Unknown |
| HQ456252 | COMJ | NA | NA | Unknown |
| HQ456253 | Com125 | NA | NA | Unknown |
| HQ456254 | KPA15 | NA | NA | Unknown |
| HQ456255 | Lamu33 | NA | NA | Unknown |
| HQ846356 | GD115 | China | 2010_10 | Human |
| HQ846357 | GD113 | China | 2010_10 | Human |
| HQ846358 | GD139 | China | 2010_10 | Human |
| HQ846359 | GD134 | China | 2010_10 | Human |
| HW249775 | UNKNOWN_HW249775 | NA | NA | Unknown |
| HW249776 | UNKNOWN_HW249776 | NA | NA | Unknown |
| HW249777 | UNKNOWN_HW249777 | NA | NA | Unknown |
| HW249778 | UNKNOWN_HW249778 | NA | NA | Unknown |
| HW249779 | UNKNOWN_HW249779 | NA | NA | Unknown |
| HW249780 | UNKNOWN_HW249780 | NA | NA | Unknown |
| HW642747 | UNKNOWN_HW642747 | NA | NA | Unknown |
| HW642748 | UNKNOWN_HW642748 | NA | NA | Unknown |
| HW642749 | UNKNOWN_HW642749 | NA | NA | Unknown |
| HW642750 | UNKNOWN_HW642750 | NA | NA | Unknown |
| HW642751 | UNKNOWN_HW642751 | NA | NA | Unknown |
| HW642752 | UNKNOWN_HW642752 | NA | NA | Unknown |
| JA879127 | UNKNOWN_JA879127 | NA | NA | Unknown |
| JA879128 | UNKNOWN_JA879128 | NA | NA | Unknown |
| JA879129 | UNKNOWN_JA879129 | NA | NA | Unknown |
| JA879130 | UNKNOWN_JA879130 | NA | NA | Unknown |
| JA879131 | UNKNOWN_JA879131 | NA | NA | Unknown |
| JA879132 | UNKNOWN_JA879132 | NA | NA | Unknown |
| JB138046 | UNKNOWN_JB138046 | NA | NA | Unknown |
| JB160217 | UNKNOWN_JB160217 | NA | NA | Unknown |
| JB160218 | UNKNOWN_JB160218 | NA | NA | Unknown |
| JB160219 | UNKNOWN_JB160219 | NA | NA | Unknown |
| JB160220 | UNKNOWN_JB160220 | NA | NA | Unknown |
| JB160221 | UNKNOWN_JB160221 | NA | NA | Unknown |
| JB160222 | UNKNOWN_JB160222 | NA | NA | Unknown |
| JF274082 | IND_06_Guj | India | 2006_09_27 | Human |
| JN558834 | SVUCTR_09 | India | 2009 | Human |
| JN558835 | SVUKDP_08 | India | 2008 | Human |
| JN558836 | SVUKDP_09 | India | 2009 | Human |
| JQ065885 | DG891 | China | 2010_10 | Human |
| JQ065886 | DG892 | China | 2010_10 | Human |
| JQ065887 | DG893 | China | 2010_10 | Human |
| JQ065888 | DG894 | China | 2010_10 | Human |
| JQ065889 | DG895 | China | 2010_10 | Human |
| JQ065890 | GZ0991 | China | 2010_10 | Human |
| JQ065891 | GZ1029 | China | 2010_10 | Human |
| JQ065892 | GZ3626 | China | 2010_10 | Human |
| JQ067624 | CHI2010 | China | 2010 | Unknown |
| JQ861253 | V1024306_KH11_PVH | Cambodia | 2011_08_16 | Human |
| JQ861254 | V1024308_KH11_PVH | Cambodia | 2011_08_16 | Human |
| JQ861255 | V1024310_KH11_PVH | Cambodia | 2011_08_16 | Human |
| JQ861256 | V1024311_KH11_PVH | Cambodia | 2011_08_16 | Human |
| JQ861257 | V1024313_KH11_PVH | Cambodia | 2011_08_16 | Human |
| JQ861258 | V1024314_KH11_PVH | Cambodia | 2011_08_16 | Human |
| JQ861259 | V0603308_KH11_BTB | Cambodia | 2011_05_26 | Human |
| JQ861260 | V0603310_KH11_BTB | Cambodia | 2011_05_28 | Human |
| JX088705 | GD05_2010 | China | 2010 | Human |
| KC488650 | CHIKV_JC2012 | China | 2012 | Human |
| KC614648 | Yem_11 | Yemen | 2011_01_25 | *Aedes aegypti* |
| KC862329 | NL10_152 | Indonesia | 2010 | Human |
| KF151174 | D47 | Myanmar | 2009_07_13 | Human |
| KF151175 | D136 | Myanmar | 2009_12_11 | Human |
| KF283986 | L2 | Comoros | 2005 | Human |
| KF283987 | S7 | Comoros | 2005 | Human |
| KF318729 | chik_sy | China | 2012_07_06 | Human |
| KF590564 | 10Mdy7 | Myanmar | 2010 | Human |
| KF590565 | 10Mdy20 | Myanmar | 2010 | Human |
| KF590566 | 10Mdy30 | Myanmar | 2010 | Human |
| KF872195 | LEIV_CHIKV_Moscow_1_2013 | Russia | 2013_09_24 | Human |
| KJ451622 | 3807 | Micronesia | 2013_10 | Human |
| KJ451623 | 3462 | Micronesia | 2013_10 | Human |
| KJ451624 | 99659 | British Virgin Islands | 2014_01 | Human |
| KJ579184 | BK46 | Thailand | 2013_10_14 | Human |
| KJ579185 | BK57 | Thailand | 2013_10_14 | Human |
| KJ579186 | BK63 | Thailand | 2013_10_14 | Human |
| KJ579187 | BK68 | Thailand | 2013_10_14 | Human |
| KJ679577 | CHIKV_STMWG01 | India | 2011_09_12 | Human |
| KJ679578 | CHIKV_STMWG02 | India | 2011_12_21 | Human |
| KJ689452 | Yap_13_2039 | Micronesia | 2013_11 | Unknown |
| KJ689453 | Yap_13_2148 | Micronesia | 2013_11 | Unknown |
| KJ796844 | RGCB730_09 | India | 2009_08_04 | Unknown |
| KJ796845 | RGCB729_09 | India | 2009_08_04 | Unknown |
| KJ796846 | RGCB755_09 | India | 2009_08_18 | Unknown |
| KJ796847 | CK650 | Thailand | 2009_03_05 | Unknown |
| KJ796848 | CK80 | Thailand | 2008_11_06 | Unknown |
| KJ796849 | CK233 | Thailand | 2008_12_23 | Unknown |
| KJ796850 | CK11_53 | Thailand | 2009_09_16 | Unknown |
| KJ796851 | CK257 | Thailand | 2008_12_01 | Unknown |
| KJ796852 | CK539 | Thailand | 2009_02_12 | Unknown |
| KJ941050 | R91064 | USA | 2006 | Human |
| KM673291 | DH130003 | Indonesia | 2013_01 | Human |
| KM923917 | M125 | Malaysia | 2007_03_09 | Monkey |
| KM923918 | M127 | Malaysia | 2007_03_09 | Monkey |
| KM923919 | M128 | Malaysia | 2007_03_09 | Monkey |
| KM923920 | M129 | Malaysia | 2007_03_09 | Monkey |
| KP003807 | OPY6 | France | 2006 | Human |
| KP003808 | MADOPY1 | Madagascar | 2006 | Human |
| KP003809 | OPY4 | Mayotte | 2006 | Human |
| KP003810 | StVE | Italy | 2007 | Human |
| KP003811 | StBI | Italy | 2007 | Human |
| KP003812 | GABOPY1 | Gabon | 2007 | Human |
| KP003813 | BRAZZA_MRS1 | Republic of the Congo | 2011 | Human |
| KP164567 | AMA2798_H804298 | Brazil_Amapa_Oiapoque | 2014_08_28 | Human |
| KP164568 | BHI3734_H804698 | Brazil_Bahia_Feira de Santana | 2014_08_26 | Human |
| KP164569 | BHI3741_H804705 | Brazil_Bahia_Feira de Santana | 2014_08_28 | Human |
| KP164570 | BHI3745_H804709 | Brazil_Bahia_Feira de Santana | 2014_09_03 | Human |
| KP164571 | PER160_H803609 | Brazil_Pernambuco_Recife | 2014_07_03 | Human |
| KP164572 | TR206_H804187 | Brazil_Para_Belem | 2014_08_21 | Human |
| KP164869 | 25 | Thailand | 2009 | Unknown |
| KP702297 | Com5 | Comoros | 2005 | Human |
| KP851709 | InDRE_51CHIK | Mexico | 2014_10_15 | Human |
| KP851710 | InDRE_4CHIK | Mexico | 2014_05_30 | Human |
| KR046227 | VE53_20 | Trinidad and Tobago | 2014_08_30 | Human |
| KR046228 | VE54_19 | Trinidad and Tobago | 2014_09_11 | Human |
| KR046229 | VE56_9 | Trinidad and Tobago | 2014_09_17 | Human |
| KR046230 | VE56_13 | Trinidad and Tobago | 2014_09_18 | Human |
| KR046231 | VE54_20 | Trinidad and Tobago | 2014_11_09 | Human |
| KR046232 | VE56_20 | Trinidad and Tobago | 2014_09_20 | Human |
| KR046233 | VE57_2 | Trinidad and Tobago | 2014_09_20 | Human |
| KR046234 | VE55_4 | Trinidad and Tobago | 2014_09_12 | Human |
| KR264949 | PR_S4 | Puerto Rico | 2014_07_15 | Human |
| KR264950 | PR_S5 | Puerto Rico | 2014_07_16 | Human |
| KR264951 | PR_S6 | Puerto Rico | 2014_08_14 | Human |
| KR559470 | WHCHK1 | USA | 2014_11 | Unknown |
| KR559471 | WHCHK2 | El Salvador | 2014_10 | Unknown |
| KR559472 | WHCHK3 | El Salvador | 2014_06 | Unknown |
| KR559473 | WHCHK4 | French Polynesia | 2015_02 | Unknown |
| KR559474 | WHCHK5 | USA | 2014_09 | Unknown |
| KR559475 | WHCHK6 | El Salvador | 2014_09 | Unknown |
| KR559476 | WHCHK7 | Haiti | 2014_07 | Unknown |
| KR559477 | WHCHK8 | Dominican Republic | 2014_07 | Unknown |
| KR559478 | WHCHK9 | Haiti | 2014_05 | Unknown |
| KR559479 | WHCHK10 | Dominican Republic | 2014_04 | Unknown |
| KR559480 | WHCHK11 | USA | 2014_09 | Unknown |
| KR559481 | WHCHK12 | Guatemala | 2014_09 | Unknown |
| KR559482 | WHCHK13 | USA | 2014_08 | Unknown |
| KR559483 | WHCHK14 | USA | 2014_10 | Unknown |
| KR559484 | WHCHK15 | El_Salvador | 2014_11 | Unknown |
| KR559485 | WHCHK16 | USA | 2014_10 | Unknown |
| KR559486 | WHCHK17 | Panama | 2014_11 | Unknown |
| KR559487 | WHCHK18 | Honduras | 2014_07 | Unknown |
| KR559488 | WHCHK19 | Honduras | 2014_09 | Unknown |
| KR559489 | WHCHK20 | Jamaica | 2014_10 | Unknown |
| KR559490 | WHCHK21 | Guyana | 2014_08 | Unknown |
| KR559491 | WHCHK22 | Colombia | 2014_08 | Unknown |
| KR559492 | WHCHK23 | Saint Lucia | 2014_08 | Unknown |
| KR559493 | WHCHK24 | NA | 2014 | Unknown |
| KR559494 | WHCHK25 | USA | 2014_07 | Unknown |
| KR559495 | WHCHK26 | USA | 2014_07 | Unknown |
| KR559496 | WHCHK27 | Guyana | 2014_07 | Unknown |
| KR559497 | WHCHK28 | NA | 2014_06 | Unknown |
| KR559498 | WHCHK29 | Dominican Republic | 2014_03 | Unknown |
| KT192707 | 11540 | Nicaragua | 2014_10_31 | Human |
| KT308159 | CPCC007800Y01 | Philippines | 2012 | Human |
| KT308160 | CPCC017800Y01 | Philippines | 2012 | Human |
| KT308161 | CPCC065200Y01 | Philippines | 2012 | Human |
| KT308162 | CPCC083400Y01 | Philippines | 2012 | Human |
| KT308163 | CPCC095700Y01 | Philippines | 2012 | Human |
| KT324224 | MY_08_1230 | Malaysia | 2008_12 | Human |
| KT324225 | MY_09_4493 | Malaysia | 2009_01 | Human |
| KT324226 | MY_09_5668 | Malaysia | 2009_01 | Human |
| KT324227 | MY_09_3926 | Malaysia | 2009_03 | Human |
| KT324228 | MY_09_5559 | Malaysia | 2009_02 | Human |
| KT327163 | CH0008 | Mexico | 2014 | Human |
| KT327164 | CH0045 | Mexico | 2014 | Human |
| KT327165 | CH0072 | Mexico | 2014 | Human |
| KT327166 | LI0031 | Mexico | 2014 | Human |
| KT327167 | TA0006 | Mexico | 2014 | Human |
| KT336777 | RGCB711_09 | India | 2009_04_08 | Unknown |
| KT336778 | RGCB855_10 | India | 2010_07_16 | Unknown |
| KT336779 | RGCB1259_12 | India | 2012_08_03 | Unknown |
| KT336780 | RGCB1264_12 | India | 2012_08_03 | Unknown |
| KT336781 | RGCB1356_13 | India | 2013_03_06 | Unknown |
| KT336782 | RGCB1431_13 | India | 2013_05_27 | Unknown |
| KT449801 | LR2006_OPY1 | Reunion | NA | Human |
| KT581023 | BzH1 | Brazil_Bahia | 2014 | Human |
| KU355832 | RJ_CHIKV_2015 | Brazil_RJ_RJ | 2015 | Human |
| KU365366 | CH_0008 | Mexico | 2014_10_09 | Human |
| KU365367 | CH_0072 | Mexico | 2014_11_07 | Human |
| KU365368 | TA_0006 | Mexico | 2014_03_26 | Human |
| KU365369 | VE56_13 | Trinidad and Tobago | 2014 | Human |
| KU365370 | DOH013 | Bangladesh | 2011_11 | Human |
| KU365371 | CS0056 | Bangladesh | 2011_11 | Human |
| KU365372 | 241 | Colombia | 2014_12_18 | Human |
| KU365373 | 246 | Colombia | 2014_12 | Human |
| KU365374 | 257263 | Venezuela | 2014_12_17 | Human |
| KU940225 | Bahia08 | Brazil_Bahia | 2015_07_15 | Human |
| KX009167 | Chik435 | Thailand | 2013 | Human |
| KX009168 | Chik409 | Thailand | 2013 | Human |
| KX009169 | Chik406 | Thailand | 2013 | Human |
| KX009170 | Chik398 | Thailand | 2013 | Human |
| KX009171 | Chik397 | Thailand | 2013 | Human |
| KX097982 | JMB_154 | Indonesia | 2015 | Human |
| KX097986 | JMB_192 | Indonesia | 2015 | Human |
| KX097988 | JMB_230 | Indonesia | 2015 | Human |
| KX168429 | BR33 | Brazil_Pernambuco | 2016_03_03 | Human |
| KX228391 | BR33 | Brazil_Pernambuco | 2016_03_03 | Human |
| KX262986 | Aedes_furcifer_37937 | Senegal | 1983 | Mosquito |
| KX262987 | CHIKV_THA_SVO_451_96_1996 | Thailand | 1996 | Human |
| KX262988 | 6307_88_1988 | Thailand | 1988 | Human |
| KX262989 | CHIKV_ITA_Bianchi_2007 | Italy | 2007 | Human |
| KX262990 | 23161 | NA | 1959 | Unknown |
| KX262991 | SXM_H_20235 | Saint Martin | 2003 | Human |
| KX262992 | CHIKV_GLP_YO_111213_2014 | Guadeloupe | 2014_01_05 | Human |
| KX262993 | CHIKV_ITA_Venturini_2007 | Italy | 2007 | Human |
| KX262994 | CHIKV_GUF_YO_123223_2014 | French Guiana | 2014_01_21 | Human |
| KX262995 | Aedes_furcifer_37950 | Senegal | 1983_07_10 | Mosquito |
| KX262996 | CHIKV_CMR_667_2006 | Cameroon | 2006 | Human |
| KX262997 | MYS_BS_285_C2 | Malaysia | 2009 | Human |
| KX496989 | UF_1_2016 | Colombia | 2016_02_09 | Human |
| KX619422 | HUBCK18 | India | 2014_07_08 | Human |
| KX619423 | HUBCK49 | India | 2014_07_23 | Human |
| KX619424 | JHCK96 | India | 2015_09_22 | Human |
| KX619425 | JHCK87 | India | 2015_09_11 | Human |
| KX619426 | JHCK128 | India | 2015_12_03 | Human |
| KX702401 | 1_2014 | Haiti | 2014_06_02 | Human |
| KX702402 | 2_2014 | Haiti | 2014_06_09 | Human |
| KX881784 | APCK406 | India | NA | Human |
| KY038946 | ArB6445 | Central African Republic | 1975_06 | *Aedes opok* |
| KY038947 | HB84P07 | Central African Republic | 1983_12 | Human |
| KY055011 | C302F | Brazil_Sergipe_Aracaju | 2016_02_20 | Mosquito |
| KY057363 | 119067 | India | 2016_08_28 | Human |
| KY124328 | RJ_IB1 | Brazil_RJ_RJ | 2016_03_16 | Human |
| KY124329 | RJ_IB5 | Brazil_RJ_RJ | 2016_03_16 | Human |
| KY272961 | N581 | Dominican Republic | 2014 | Human |
| KY272962 | N594 | Dominican Republic | 2014 | Human |
| KY272963 | N494 | Dominican Republic | 2014 | Human |
| KY272964 | N493 | Dominican Republic | 2014 | Human |
| KY272965 | N490 | Dominican Republic | 2014 | Human |
| KY272966 | N469 | Dominican Republic | 2014 | Human |
| KY272967 | N468 | Dominican Republic | 2014 | Human |
| KY272968 | N424 | Dominican Republic | 2014 | Human |
| KY272969 | N300 | Dominican Republic | 2014 | Human |
| KY272970 | N587 | Dominican Republic | 2014 | Human |
| KY415978 | Haiti_3_2014 | Haiti | 2014_05_29 | Human |
| KY415979 | Haiti_4_2014 | Haiti | 2014_05_29 | Human |
| KY415980 | Haiti_5_2014 | Haiti | 2014_06_05 | Human |
| KY415981 | Haiti_6_2014 | Haiti | 2014_06_10 | Human |
| KY415982 | Haiti_7_2014 | Haiti | 2014_06_11 | Human |
| KY415983 | Haiti_8_2014 | Haiti | 2014_06_02 | Human |
| KY415984 | Haiti_9_2014 | Haiti | 2014_06_24 | Human |
| KY415985 | Haiti_10_2014 | Haiti | 2014_08_13 | Human |
| KY435454 | 14_06638 | Trinidad and Tobago | 2011_11 | Human |
| KY435455 | 14_06523 | Anguilla | 2014_11_12 | Human |
| KY435456 | 14_06350 | Suriname | 2014_08_17 | Human |
| KY435457 | 14_06252 | Montserrat | 2014_10_30 | Human |
| KY435458 | 14_06121 | Guyana | 2014_11_03 | Human |
| KY435459 | 14_05085 | Cayman Islands | 2014_09_17 | Human |
| KY435460 | 14_05081 | Cayman Islands | 2014_07_06 | Human |
| KY435461 | 14_04561 | Jamaica | 2014_08_24 | Human |
| KY435462 | 14_04558 | Jamaica | 2014_08_25 | Human |
| KY435463 | 14_04444 | Suriname | 2014_08_02 | Human |
| KY435464 | 14_04425 | Barbados | 2014_08_15 | Human |
| KY435465 | 14_04279 | Trinidad and Tobago | 2014_08_17 | Human |
| KY435466 | 14_03985 | Barbados | 2014_08_06 | Human |
| KY435467 | 14_03844 | Montserrat | 2014_07_24 | Human |
| KY435468 | 14_03837 | Jamaica | 2014_08_06 | Human |
| KY435469 | 14_03562 | Grenada | 2014_07_30 | Human |
| KY435470 | 14_02961 | Bahamas | 2014_07_08 | Human |
| KY435471 | 14_02585 | Turks and Caicos Islands | 2014_06_11 | Human |
| KY435472 | 14_02560 | Grenada | 2014_06_16 | Human |
| KY435473 | 14_02557 | Saint Lucia | 2014_05_22 | Human |
| KY435474 | 14_02526 | Saint Lucia | 2014_05_19 | Human |
| KY435475 | 14_02346 | Saint Vincent and the Grenadines | 2014_05_22 | Human |
| KY435476 | 14_02306 | Turks and Caicos Islands | 2014_06_05 | Human |
| KY435477 | 14_02217 | Guyana | 2014_05_31 | Human |
| KY435478 | 14_02086 | Guyana | 2014_05_17 | Human |
| KY435479 | 14_01526 | Antigua and Barbuda | 2014_04_28 | Human |
| KY435480 | 14_01507 | Haiti | 2014_04_27 | Human |
| KY435481 | 14_01349 | Saint Lucia | 2014_04_22 | Human |
| KY435482 | 14_01152 | Saint Kitts and Nevis | 2014_03_11 | Human |
| KY435483 | 14_00686 | Anguilla | 2014_02_12 | Human |
| KY435484 | 14_00448 | Dominica | 2014_01_30 | Human |
| KY435485 | 14_00324 | Dominica | 2014_01_28 | Human |
| KY435486 | 14_00309 | British Virgin Islands | 2014_01_23 | Human |
| KY575565 | IDR1400024561 | USA | 2014 | Human |
| KY575566 | IDR1400019200_2014 | USA | 2014 | Human |
| KY575567 | 91077_2006 | USA | 2006 | Human |
| KY575568 | 91064A_2006 | USA | 2006 | Human |
| KY575569 | IDR1400021488 | USA | 2014 | Human |
| KY575570 | 2008_Sri Lanka_2008 | USA | 2008 | Human |
| KY575571 | La_Reunion_OYP1_2006 | USA | 2006 | Human |
| KY575572 | IDR1400023906_2014 | USA | 2014 | Human |
| KY575573 | IDR1400021177 | USA | 2014 | Human |
| KY575574 | HIMSTSSA287_1995 | USA | 1995 | Human |
| KY680347 | CKVHL_47_2014 | USA | 2014_05_13 | Human |
| KY680348 | CKVHL_80 | USA | 2014_09_05 | Human |
| KY680349 | CKVHL_97_2014 | USA | 2014_07_02 | Human |
| KY680350 | CKVHL_61 | USA | 2014_12_10 | Human |
| KY680351 | CKVHL_26_2014 | USA | 2014_06_24 | Human |
| KY680352 | CKVHL_126_2014 | USA | 2014_05_15 | Human |
| KY680353 | CKVHL_90_2014 | USA | 2014_08_25 | Human |
| KY680354 | CKVHL_32_2014 | USA | 2014_05_08 | Human |
| KY680355 | CKVHL_125_2014 | USA | 2014_07_22 | Human |
| KY680356 | CKVHL_30_2014 | USA | 2014_07_21 | Human |
| KY680357 | CKVHL_51_2014 | USA | 2014_08_09 | Human |
| KY680358 | 33 | USA | 2014_08_08 | Human |
| KY680359 | CKVHL_29_2014 | USA | 2014_07_04 | Human |
| KY680360 | CKVHL_114_2014 | USA | 2014_08_05 | Human |
| KY680361 | CKVHL_136_2014 | USA | 2014_05_26 | Human |
| KY680362 | CKVHL_81_2014 | USA | 2014_07_26 | Human |
| KY680363 | CKVHL_111_2014 | USA | 2014_06_15 | Human |
| KY680364 | CKVHL_139_2014 | USA | 2014_05_15 | Human |
| KY680365 | CKVHL_66 | USA | 2014_09_18 | Human |
| KY680366 | CKVHL_10_2015 | USA | 2015_07_24 | Human |
| KY680367 | CKVHL_49_2014 | USA | 2014_06_09 | Human |
| KY680368 | CKVHL_115_2014 | USA | 2014_12_01 | Human |
| KY680369 | CKVHL_22_2014 | USA | 2014_05_24 | Human |
| KY680370 | CKVHL_57_2014 | USA | 2014_10_03 | Human |
| KY680371 | CKVHL_95_2014 | USA | 2014_08_20 | Human |
| KY680372 | CKVHL_86_2014 | USA | 2014_06_04 | Human |
| KY680373 | CKVHL_113_2014 | USA | 2014_10_22 | Human |
| KY680374 | CKVHL_89_2014 | USA | 2014_12_06 | Human |
| KY680375 | CKVHL_24_2014 | USA | 2014_06_04 | Human |
| KY680376 | CKVHL_85_2014 | USA | 2014_10_08 | Human |
| KY680377 | CKVHL_28_2014 | USA | 2014_08_16 | Human |
| KY680378 | CKVHL_103_2014 | USA | 2014_10_19 | Human |
| KY680379 | CKVHL_53_2014 | USA | 2014_09_16 | Human |
| KY680380 | CKVHL_104_2014 | USA | 2014_10_13 | Human |
| KY680381 | CKVHL_79_2014 | USA | 2014_10_21 | Human |
| KY680382 | CKVHL_135_2014 | USA | 2014_06_04 | Human |
| KY680383 | CKVHL_121_2014 | USA | 2014_08_20 | Human |
| KY680384 | CKVHL_03_2014 | USA | 2014_06_25 | Human |
| KY680385 | CKVHL_70_2014 | USA | 2014_11_25 | Human |
| KY680386 | CKVHL_16_2014 | USA | 2014_09_24 | Human |
| KY680387 | CKVHL_18_2014 | USA | 2014_10_14 | Human |
| KY680388 | CKVHL_76 | USA | 2014_10_02 | Human |
| KY680389 | CKVHL_59_2015 | USA | 2015_07_14 | Human |
| KY680390 | CKVHL_23_2014 | USA | 2014_05_07 | Human |
| KY680391 | CKVHL_72_2014 | USA | 2014_10_24 | Human |
| KY680392 | CKVHL_09_2014 | USA | 2014_10_19 | Human |
| KY680393 | CKVHL_42_2014 | USA | 2014_09_17 | Human |
| KY680394 | CKVHL_99 | USA | 2014_10_05 | Human |
| KY680395 | CKVHL_08 | USA | 2014_07_21 | Human |
| KY680396 | CKVHL_35_2014 | USA | 2014_09_02 | Human |
| KY680397 | CKVHL_01_2014 | USA | 2014_05_23 | Human |
| KY680398 | CKVHL_119_2014 | USA | 2014_08_05 | Human |
| KY680399 | CKVHL_120_2014 | USA | 2014_09_05 | Human |
| KY680400 | CKVHL_27_2014 | USA | 2014_07_15 | Human |
| KY680401 | CKVHL_101_2014 | USA | 2014_09_22 | Human |
| KY680402 | CKVHL_82_2014 | USA | 2014_07_27 | Human |
| KY680403 | CKVHL_91_2014 | USA | 2014_06_14 | Human |
| KY680404 | CKVHL_67_2014 | USA | 2014_09_17 | Human |
| KY680405 | CKVHL_96_2014 | USA | 2014_06_18 | Human |
| KY680406 | CKVHL_05_2014 | USA | 2014_08_08 | Human |
| KY680407 | CKVHL_116_2014 | USA | 2014_11_20 | Human |
| KY680408 | CKVHL_34_2014 | USA | 2014_06_26 | Human |
| KY680409 | CKVHL_124_2014 | USA | 2014_10_21 | Human |
| KY680410 | CKVHL_20_2014 | USA | 2014_08_22 | Human |
| KY680411 | CKVHL_94_2014 | USA | 2014_09_04 | Human |
| KY680412 | CKVHL_88 | USA | 2014_08_28 | Human |
| KY680413 | CKVHL_128_2014 | USA | 2014_09_17 | Human |
| KY680414 | CKVHL_21_2014 | USA | 2014_09_13 | Human |
| KY703888 | 1885_1D_2015 | Nicaragua | 2015_08_15 | Human |
| KY703889 | 1863_1C_2015 | Nicaragua | 2015_08_05 | Human |
| KY703890 | 11132_15_2015 | Nicaragua | 2015_09_23 | Human |
| KY703891 | 11006_15_2015 | Nicaragua | 2015_09_17 | Human |
| KY703892 | 1988_1C_2015 | Nicaragua | 2015_11_26 | Human |
| KY703893 | 1908_1C_2015 | Nicaragua | 2015_09_02 | Human |
| KY703894 | 6446_1LA1_2015 | Nicaragua | 2015_10_09 | Human |
| KY703895 | 1346_12_A_1_2015 | Nicaragua | 2015_11_26 | Human |
| KY703896 | 1816_1E | Nicaragua | 2014_12_18 | Human |
| KY703897 | 1825_1C_2015 | Nicaragua | 2015_01_16 | Human |
| KY703898 | 1897_1C_2015 | Nicaragua | 2015_08_25 | Human |
| KY703899 | 12438_15_2015 | Nicaragua | 2015_10_20 | Human |
| KY703900 | 1993_1C_2015 | Nicaragua | 2015_12_14 | Human |
| KY703901 | 1838_1C_2015 | Nicaragua | 2015_01_26 | Human |
| KY703902 | CKVGH11_2015 | Nicaragua | 2015_09_05 | Human |
| KY703903 | 1912_1C_2015 | Nicaragua | 2015_09_05 | Human |
| KY703904 | 5823_11A1_2014 | Nicaragua | 2014_10_04 | Human |
| KY703905 | 1920_1C | Nicaragua | 2015_09_17 | Human |
| KY703906 | 1914_1C_2015 | Nicaragua | 2015_09_08 | Human |
| KY703907 | 996_12_A_1_2015 | Nicaragua | 2015_07_15 | Human |
| KY703908 | 6826_11A1_2014 | Nicaragua | 2014_12_02 | Human |
| KY703909 | 1842_1C_2015 | Nicaragua | 2015_01_28 | Human |
| KY703910 | 12062_15_2015 | Nicaragua | 2015_10_18 | Human |
| KY703911 | 1167_12_A_1_2015 | Nicaragua | 2015_12_17 | Human |
| KY703912 | 15388_15 | Nicaragua | 2015_12_14 | Human |
| KY703913 | 1878_1C_2015 | Nicaragua | 2015_08_13 | Human |
| KY703914 | 1886_1D_2015 | Nicaragua | 2015_08_15 | Human |
| KY703915 | 1857_1C_2015 | Nicaragua | 2015_08_03 | Human |
| KY703916 | 6556_12_A_1_2015 | Nicaragua | 2015_08_04 | Human |
| KY703917 | 14_16_2015 | Nicaragua | 2016_01_08 | Human |
| KY703918 | 1839_1C_2015 | Nicaragua | 2015_01_26 | Human |
| KY703919 | 1844_1C_2015 | Nicaragua | 2015_01_29 | Human |
| KY703920 | 13491_15 | Nicaragua | 2015_11_16 | Human |
| KY703921 | 1967_1C_2015 | Nicaragua | 2015_11_04 | Human |
| KY703922 | 7074_12A1_2015 | Nicaragua | 2015_09_06 | Human |
| KY703923 | 1925_1C_2015 | Nicaragua | 2015_09_19 | Human |
| KY703924 | 1879_1C_2015 | Nicaragua | 2015_08_13 | Human |
| KY703925 | 5201_12A1 | Nicaragua | 2015_09_06 | Human |
| KY703926 | 1891_1C_2015 | Nicaragua | 2015_08_20 | Human |
| KY703927 | 1822_1C_2015 | Nicaragua | 2015_01_15 | Human |
| KY703928 | 1847_1C_2015 | Nicaragua | 2015_01_30 | Human |
| KY703930 | 4912_12_A_1_2015 | Nicaragua | 2015_11_24 | Human |
| KY703931 | 1924_1C_2015 | Nicaragua | 2015_09_19 | Human |
| KY703932 | 1890_1D_2015 | Nicaragua | 2015_08_19 | Human |
| KY703933 | 8476_1LA1_2015 | Nicaragua | 2015_10_09 | Human |
| KY703934 | 8065_1LA1_2015 | Nicaragua | 2015_10_27 | Human |
| KY703935 | 4900_12_A | Nicaragua | 2015_12_02 | Human |
| KY703936 | 1497_1LA1_2015 | Nicaragua | 2015_10_07 | Human |
| KY703937 | 1956_1C_2015 | Nicaragua | 2015_10_15 | Human |
| KY703938 | 1852_1D_2015 | Nicaragua | 2015_02_18 | Human |
| KY703939 | 14577_15_2015 | Nicaragua | 2015_12_01 | Human |
| KY703940 | 1756_1C_2014 | Nicaragua | 2014_10_28 | Human |
| KY703941 | 12687_15 | Nicaragua | 2015_10_28 | Human |
| KY703942 | 86_12_A_1_2015 | Nicaragua | 2015_08_04 | Human |
| KY703943 | 1948_1C_2015 | Nicaragua | 2015_10_10 | Human |
| KY703944 | 1938_1C_2015 | Nicaragua | 2015_10_03 | Human |
| KY703945 | 1937_1C | Nicaragua | 2015_10_02 | Human |
| KY703946 | 1952_1C | Nicaragua | 2015_10_12 | Human |
| KY703947 | 1778_1C_2014 | Nicaragua | 2014_11_19 | Human |
| KY703948 | 1889_1D_2015 | Nicaragua | 2015_08_18 | Human |
| KY703949 | 1824_1C_2015 | Nicaragua | 2015_01_15 | Human |
| KY703950 | 1790_1C_2014 | Nicaragua | 2014_11_25 | Human |
| KY703951 | 5883_12A1 | Nicaragua | 2015_08_05 | Human |
| KY703952 | CKVGH13 | Nicaragua | 2015_09_03 | Human |
| KY703954 | 1758_1C_2014 | Nicaragua | 2014_11_05 | Human |
| KY703955 | 2741_12_A_1_2015 | Nicaragua | 2015_08_17 | Human |
| KY703956 | 7172_11A1_2014 | Nicaragua | 2014_10_28 | Human |
| KY703957 | 7555_12A1_2015 | Nicaragua | 2015_09_05 | Human |
| KY703958 | 1985_1C_2015 | Nicaragua | 2015_11_25 | Human |
| KY703959 | 1800_1D_2014 | Nicaragua | 2014_12_03 | Human |
| KY703960 | 1864_1C_2015 | Nicaragua | 2015_08_05 | Human |
| KY703961 | 7101_12_A__2015 | Nicaragua | 2015_08_06 | Human |
| KY703962 | 11630_15_2015 | Nicaragua | 2015_10_01 | Human |
| KY703963 | 1913_1C_2015 | Nicaragua | 2015_09_07 | Human |
| KY703965 | 1909_1C_2015 | Nicaragua | 2015_09_03 | Human |
| KY703966 | 4036_12 | Nicaragua | 2015_12_10 | Human |
| KY703967 | 8976_12_A_1_2015 | Nicaragua | 2015_08_12 | Human |
| KY703968 | 1837_1D_2015 | Nicaragua | 2015_01_23 | Human |
| KY703969 | 1773_1C_2014 | Nicaragua | 2014_11_14 | Human |
| KY703970 | 14905_15_2015 | Nicaragua | 2015_11_28 | Human |
| KY703971 | 14906_15_2015 | Nicaragua | 2015_11_28 | Human |
| KY703972 | 1805_1E | Nicaragua | 2014_12_09 | Human |
| KY703973 | 6523_12_A_1_2015 | Nicaragua | 2015_11_06 | Human |
| KY703974 | 1910_1C_2015 | Nicaragua | 2015_09_03 | Human |
| KY703975 | 11091_15_2015 | Nicaragua | 2015_09_22 | Human |
| KY703976 | 6450_12A1 | Nicaragua | 2015_09_05 | Human |
| KY703977 | 1835_12A1_2015 | Nicaragua | 2015_08_03 | Human |
| KY703978 | 14648_15_2015 | Nicaragua | 2015_12_02 | Human |
| KY703979 | 11093_15_2015 | Nicaragua | 2015_09_22 | Human |
| KY703980 | 6638_12A1_2015 | Nicaragua | 2015_09_06 | Human |
| KY703982 | 4040_12_A_1_2015 | Nicaragua | 2015_11_30 | Human |
| KY703983 | 1809_1E_2014 | Nicaragua | 2014_12_11 | Human |
| KY703984 | 1794_1C_2014 | Nicaragua | 2014_11_27 | Human |
| KY703985 | 1880_1D_2015 | Nicaragua | 2015_08_13 | Human |
| KY703986 | 1918_1C | Nicaragua | 2015_09_16 | Human |
| KY703987 | 1367_12_A_1_2015 | Nicaragua | 2015_11_04 | Human |
| KY703988 | 1793_1D_2014 | Nicaragua | 2014_11_27 | Human |
| KY703989 | 1802_1D | Nicaragua | 2014_12_03 | Human |
| KY703990 | 1983_1C_2015 | Nicaragua | 2015_11_21 | Human |
| KY703991 | 24044_1LA1_2015 | Nicaragua | 2015_10_09 | Human |
| KY703992 | 5695_12A1 | Nicaragua | 2015_08_05 | Human |
| KY703993 | 1760_1C_2014 | Nicaragua | 2014_11_07 | Human |
| KY703994 | 13724_15_2015 | Nicaragua | 2015_11_18 | Human |
| KY703995 | 1882_1D_2015 | Nicaragua | 2015_08_13 | Human |
| KY703996 | 5024_12_A_1_2015 | Nicaragua | 2015_12_26 | Human |
| KY703997 | 4354_12 | Nicaragua | 2015_12_19 | Human |
| KY703998 | 7156_12A1_2015 | Nicaragua | 2015_08_06 | Human |
| KY703999 | 1828_1C_2015 | Nicaragua | 2015_01_19 | Human |
| KY704000 | 1862_1C_2015 | Nicaragua | 2015_08_05 | Human |
| KY704001 | 1829_1C_2015 | Nicaragua | 2015_01_21 | Human |
| KY704002 | 1823_1C_2015 | Nicaragua | 2015_01_15 | Human |
| KY704933 | 6AL | Brazil_Alagoas_Maceio | 2016_03_30 | Human |
| KY704939 | 166 | Brazil_Alagoas_Maceio | 2016_04_17 | Human |
| KY704942 | 172 | Brazil_Alagoas_Maceio | 2016_04_16 | Human |
| KY704943 | 175 | Brazil_Alagoas_Maceio | 2016_04_19 | Human |
| KY704947 | 190 | Brazil_Alagoas_Maceio | 2016_04_15 | Human |
| KY704952 | 241 | Brazil_Alagoas_Maceio | 2016_04_07 | Human |
| KY704954 | 19AP | Brazil_Paraiba_Joao Pessoa | 2016_06_20 | Human |
| KY704955 | 35AP | Brazil_Paraiba_Joao Pessoa | 2016_06_17 | Human |
| KY751908 | IN16C1 | Australia | 2016 | Human |
| KY883764 | SGEHICH02971Y13 | Singapore | 2013_01 | Human |
| L37661 | TSI_GSD_218 | USA | NA | Unknown |
| LC259082 | BaH306_NIID | Thailand | 1958 | Human |
| LC259083 | Hu_NIID58_2009 | Indonesia | 2009_09_11 | Human |
| LC259084 | Hu_NIID165 | Philippines | 2012_09_25 | Human |
| LC259085 | Hu_NIID181_2012 | Indonesia | 2012_10_15 | Human |
| LC259086 | Hu_NIID108_2013 | Indonesia | 2013_07_01 | Human |
| LC259087 | Hu_NIID112_2013 | Indonesia | 2013_07_06 | Human |
| LC259088 | Hu_NIID41 | Tonga | 2014_03_25 | Human |
| LC259089 | Hu_NIID73 | Dominica | 2014_06_23 | Human |
| LC259090 | Hu_NIID02 | Colombia | 2015_01_06 | Human |
| LC259091 | Hu_NIID35_2015 | Indonesia | 2015_05_07 | Human |
| LC259092 | Hu_NIID25 | Cuba | 2016_02_28 | Human |
| LC259093 | Hu_NIID02 | Malaysia | 2009_01_06 | Human |
| LC259094 | Hu_NIID54_2016 | Angola | 2016_05_11 | Human |
| LC331252 | 3216DP0112 | Japan | 2016 | Human |
| LC500215 | 15801056 | Aruba | 2015_01 | Human |
| LC500216 | 15801125 | Aruba | 2015_01 | Human |
| LC500217 | 15801136 | Aruba | 2015_01 | Human |
| LC500218 | 15801160 | Aruba | 2015_01 | Human |
| LC500219 | 15801358 | Aruba | 2015_01 | Human |
| LC500220 | 15801567 | Aruba | 2015_01 | Human |
| LC500221 | 15801654 | Aruba | 2015_02 | Human |
| LC500222 | 15802650 | Aruba | 2015_03 | Human |
| LN898093 | Caribbean | Martinique | 2013_12 | Human |
| LN898094 | Caribbean | Martinique | 2014_01 | Human |
| LN898095 | Caribbean | Martinique | 2014_01 | Human |
| LN898096 | Caribbean | Martinique | 2014_01 | Human |
| LN898097 | Caribbean | Martinique | 2014_01 | Human |
| LN898098 | Caribbean | Martinique | 2014_01 | Human |
| LN898099 | Caribbean | Martinique | 2014_01 | Human |
| LN898100 | Caribbean | Martinique | 2014_01 | Human |
| LN898101 | Caribbean | Martinique | 2014_01 | Human |
| LN898102 | Caribbean | Martinique | 2014_01 | Human |
| LN898103 | Caribbean | Martinique | 2014_01 | Human |
| LN898104 | Caribbean | Martinique | 2014_01 | Human |
| LN898105 | Caribbean | Martinique | 2014_01 | Human |
| LN898106 | Caribbean | Martinique | 2014_01 | Human |
| LN898107 | Caribbean | Martinique | 2014_01 | Human |
| LN898108 | Caribbean | Martinique | 2014_01 | Human |
| LN898109 | Caribbean | Martinique | 2014_01 | Human |
| LN898110 | Caribbean | Martinique | 2014_01 | Human |
| LN898111 | Caribbean | Martinique | 2014_01 | Human |
| LN898112 | Caribbean | Martinique | 2014_01 | Human |
| LP837225 | UNKNOWN_LP837225 | NA | NA | Unknown |
| LP837230 | UNKNOWN_LP837230 | NA | NA | Unknown |
| LP837639 | UNKNOWN_LP837639 | NA | NA | Unknown |
| LP837644 | UNKNOWN_LP837644 | NA | NA | Unknown |
| LP981862 | UNKNOWN_LP981862 | NA | NA | Unknown |
| LP981867 | UNKNOWN_LP981867 | NA | NA | Unknown |
| LY683325 | UNKNOWN_LY683325 | NA | NA | Unknown |
| MF001505 | JFRO_01 | USA | 2015 | Human |
| MF001506 | JFRO_02 | USA | 2015 | Human |
| MF001507 | JFRO_03 | USA | 2015 | Human |
| MF001508 | JFRO_04 | USA | 2015 | Human |
| MF001509 | JFRO_05 | USA | 2015 | Human |
| MF001510 | JFRO_06 | USA | 2015 | Human |
| MF001511 | JFRO_07 | USA | 2015 | Human |
| MF001512 | JFRO_08 | USA | 2015 | Human |
| MF001513 | JFRO_09 | USA | 2015 | Human |
| MF001514 | JFRO_10 | USA | 2015 | Human |
| MF001515 | JFRO_11 | USA | 2015 | Human |
| MF001516 | JFRO_12 | USA | 2015 | Human |
| MF001517 | JFRO_13 | USA | 2015 | Human |
| MF001518 | JFRO_14 | USA | 2015 | Human |
| MF001519 | JFRO_15 | USA | 2015 | Human |
| MF076568 | H2013_005_16 | Laos | 2013_03_28 | Human |
| MF076569 | H2013_007_16 | Laos | 2013_03_28 | Human |
| MF076570 | H2013_009_16 | Laos | 2013_03_28 | Human |
| MF076571 | H2013_013_16 | Laos | 2013_03_28 | Human |
| MF076572 | H2013_019_16 | Laos | 2013_03_12 | Human |
| MF076573 | H2012_019 | Laos | 2012_08_07 | Human |
| MF076574 | H2012_021 | Laos | 2012_08_16 | Human |
| MF076575 | H2012_028 | Laos | 2012_08_16 | Human |
| MF076576 | H2012_033 | Laos | 2012_08_31 | Human |
| MF076577 | M2012_006P | Laos | 2012_08_31 | Mosquito |
| MF499120 | hk02 | Hong Kong | 2016_09_15 | Human |
| MF503628 | hk01 | Hong Kong | 2016_08_26 | Human |
| MF580946 | S27_Petersfield | NA | NA | Unknown |
| MF740874 | Pakistan_03 | Pakistan | 2017 | Human |
| MF773559 | Samoa_2014 | Samoa | 2014 | Human |
| MF773560 | Caribbean_2014 | NA | 2014 | Human |
| MF773561 | Bali_2011 | Indonesia | 2011 | Human |
| MF773562 | Kiribati_2015 | Kiribati | 2015 | Human |
| MF773563 | Philippines_2014 | Philippines | 2014 | Human |
| MF773564 | Philippines_2016 | Philippines | 2016 | Human |
| MF773565 | ET2010 | East Timor | 2010 | Human |
| MF773566 | Bangladesh_2017 | Bangladesh | 2017 | Human |
| MF773567 | Borneo_2011 | Borneo | 2011 | Human |
| MF773568 | Malaysia_2008 | Malaysia | 2008 | Human |
| MF773569 | PNG_2013 | Papua New Guinea | 2013 | Human |
| MF774613 | Pakistan_01_2016 | Pakistan | 2016 | Human |
| MF774614 | Pakistan_04_2016 | Pakistan | 2016 | Human |
| MF774615 | Pakistan_05_2016 | Pakistan | 2016 | Human |
| MF774616 | Pakistan_06_2016 | Pakistan | 2016 | Human |
| MF774617 | Pakistan_07_2016 | Pakistan | 2016 | Human |
| MF774618 | Pakistan_09_2016 | Pakistan | 2016 | Human |
| MF774619 | Pakistan_10_2016 | Pakistan | 2016 | Human |
| MG000875 | Culex_quinquefasciatus_1 | Haiti | 2016_06_27 | *Culex quinquefasciatus* |
| MG000876 | Aedes_albopictusi_1 | Haiti | 2016_05_17 | *Aedes albopictus* |
| MG049915 | CHIKV_ITA_Lazio_INMI1_2017 | Italy | 2017 | Human |
| MG137428 | Shivane_CHIK2016 | India | 2016_06_29 | Human |
| MG208125 | H20235 | Saint Martin | 2013 | Human |
| MG280943 | Ross | Thailand | NA | Unknown |
| MG649970 | BRZ_38_RJ | Brazil_RJ_RJ | 2016_03_29 | Unknown |
| MG649971 | BRZ_18_RJ | Brazil_RJ_RJ | 2016_08_22 | Unknown |
| MG649972 | BRZ_10_RJ | Brazil_RJ_RJ | 2016_05_05 | Unknown |
| MG649973 | BRZ_37_RJ | Brazil_RJ_RJ | 2016_12_12 | Unknown |
| MG649974 | BRZ_3_RJ | Brazil_RJ_RJ | 2016_04_08 | Unknown |
| MG649975 | BRZ_4_RJ | Brazil_RJ_RJ | 2016_07_27 | Unknown |
| MG649976 | BRZ_20_RJ | Brazil_RJ_RJ | 2016_03_29 | Unknown |
| MG649977 | BRZ_1_RJ | Brazil_RJ_RJ | 2016_07_28 | Unknown |
| MG649978 | BRZ_14_RJ | Brazil_RJ_RJ | 2017_03_16 | Unknown |
| MG649979 | BRZ_8_RJ | Brazil_RJ_RJ | 2017_03 | Unknown |
| MG649980 | BRZ_7_RJ | Brazil_RJ_RJ | 2016_04_27 | Unknown |
| MG649981 | BRZ_2_RJ | Brazil_RJ_RJ | 2016_07_28 | Unknown |
| MG649982 | BRZ_12_RJ | Brazil_RJ_RJ | 2017_03_24 | Unknown |
| MG649983 | BRZ_6_RJ | Brazil_RJ_RJ | 2016_03_28 | Unknown |
| MG649984 | CHK_5_RJ | Brazil_RJ_RJ | 2015 | Unknown |
| MG649985 | CHK_7_RJ | Brazil_RJ_RJ | 2015 | Unknown |
| MG664850 | SZ1050 | China | 2010 | Human |
| MG664851 | SZ1239 | China | 2012 | Human |
| MG912993 | ZJQZ3_2017 | China | 2017_08_30 | Human |
| MG921596 | CH_R_1950 | Mexico | 2015 | Human |
| MG925665 | CHIKV_Henan001_2017 | China | 2017_12_15 | Human |
| MG967666 | Homo_sapiens_Haiti_11_2014 | Haiti | 2014_06 | Human |
| MH000700 | 2C | Brazil_Pernambuco_Recife | 2016_02_04 | Human |
| MH000701 | 3C | Brazil_Pernambuco_Recife | 2016_02_22 | Human |
| MH000702 | 4C | Brazil_Pernambuco_Recife | 2016_02_15 | Human |
| MH000703 | 5C | Brazil_Pernambuco_Recife | 2016_02_15 | Human |
| MH000704 | 6C | Brazil_Pernambuco_Recife | 2016_02_19 | Human |
| MH000705 | 7C | Brazil_Pernambuco_Recife | 2016_02_25 | Human |
| MH000706 | 10C | Brazil_Pernambuco_Recife | 2016_02_18 | Human |
| MH124570 | IND_2010_DEL_01 | India | 2010 | Human |
| MH124571 | IND_2010_DEL_03 | India | 2010 | Human |
| MH124572 | IND_2010_DEL_05 | India | 2010 | Human |
| MH124573 | IND_2010_DEL_06 | India | 2010 | Human |
| MH124574 | IND_2010_DEL_10 | India | 2010 | Human |
| MH124575 | IND_2010_DEL_13 | India | 2010 | Human |
| MH124576 | IND_2010_DEL_14 | India | 2010 | Human |
| MH124577 | IND_2010_DEL_20 | India | 2010 | Human |
| MH124578 | IND_2010_DEL_11 | India | 2010 | Human |
| MH124579 | IND_2010_DEL_12 | India | 2010 | Human |
| MH124580 | IND_2016_DEL_01 | India | 2016 | Human |
| MH124581 | IND_2016_DEL_02 | India | 2016 | Human |
| MH124582 | IND_2016_DEL_03 | India | 2016 | Human |
| MH124583 | IND_2016_DEL_11 | India | 2016 | Human |
| MH229986 | 6113879 | Mauritius | 2006 | Unknown |
| MH329293 | Car_61 | Colombia | 2014_11 | Human |
| MH329294 | OV_26 | Colombia | 2014_10_15 | Human |
| MH329295 | P1 | Colombia | 2014_11 | Human |
| MH329296 | M6 | Colombia | 2014_09_13 | Human |
| MH329297 | INS_477150 | Colombia | 2014_11 | Human |
| MH329298 | INS_449325 | Colombia | 2014_11 | Human |
| MH329299 | INS_449125 | Colombia | 2014_11 | Human |
| MH329300 | Car_128 | Colombia | 2014_11_03 | Human |
| MH329302 | Car_149 | Colombia | 2014_10_03 | Human |
| MH329303 | Car_4 | Colombia | 2014_10_01 | Human |
| MH329304 | Car_62 | Colombia | 2014_10_26 | Human |
| MH359139 | OV_7 | Colombia | 2014_10_15 | Human |
| MH359140 | Car_89 | Colombia | 2014_11_04 | Human |
| MH359141 | OV_13 | Colombia | 2014_10_15 | Human |
| MH359142 | OV_16 | Colombia | 2014_10_15 | Human |
| MH400249 | QZ0823 | China | 2017_08_23 | Human |
| MH423797 | 027_16_S43 | Kenya | 2016_05_07 | Human |
| MH423798 | 031_16_S65 | Kenya | 2016_05_08 | Human |
| MH423799 | 057_16_S15 | Kenya | 2016_05_31 | Human |
| MH423800 | 058_16_S33 | Kenya | 2016_05_31 | Human |
| MH423801 | 063_16_S44 | Kenya | 2016_05_31 | Human |
| MH423802 | 068_16_S55 | Kenya | 2016_05_31 | Human |
| MH423803 | 086_16_S25 | Kenya | 2016_05_31 | Human |
| MH423804 | 087_16_S34 | Kenya | 2016_06_01 | Human |
| MH423805 | 100_16_S56 | Kenya | 2016_06_01 | Human |
| MH423806 | 106_16_S75 | Kenya | 2016_06_01 | Human |
| MH423810 | 082_16_S16 | Kenya | 2016_05_31 | Human |
| MH507158 | CHIKV_ITA_Lazio_INMI2_2017 | Italy | 2017 | Human |
| MH670649 | Malaysia_2009 | China | 2009_11_19 | Human |
| MH754507 | Lazio_ISS_1_2017 | Italy | 2017_09_08 | *Aedes albopictus* |
| MH823663 | MT01 | Brazil_Mato Grosso_Cuiaba | 2017_01_05 | Human |
| MH823664 | MT02 | Brazil_Mato Grosso_Cuiaba | 2017_03_16 | Human |
| MH823665 | MT03 | Brazil_Mato Grosso_Cuiaba | 2017_03_16 | Human |
| MH823666 | MT04 | Brazil_Mato Grosso_Cuiaba | 2017_03_01 | Human |
| MH823667 | MT05 | Brazil_Mato Grosso_Cuiaba | 2017_03_01 | Human |
| MH823668 | MT06 | Brazil_Mato Grosso_Cuiaba | 2017_01_05 | Human |
| MK028836 | Com125 | Comoros | 2005 | *Aedes aegypti* |
| MK028837 | A_furcifer_wt_37997 | Senegal | 1983 | *Aedes furcifer* |
| MK028838 | SL15649 | Sri Lanka | 2006 | Human |
| MK028839 | 181_Clone_25 | USA | 1986 | Unknown |
| MK028840 | AF15561 | Thailand | 1962 | Human |
| MK086029 | SL_R233 | Sri Lanka | 2006_11_14 | Human |
| MK120194 | Calabria_ISS_1_2017 | Italy | 2017_09_27 | *Aedes albopictus* |
| MK120195 | Calabria_ISS_972_2017 | Italy | 2017_09_27 | Human |
| MK120196 | Calabria_ISS_989_2017 | Italy | 2017_09_30 | Human |
| MK120197 | Calabria_ISS_991_2017 | Italy | 2017_08_02 | Human |
| MK120198 | Calabria_ISS_977_2017 | Italy | 2017_08_02 | Human |
| MK120199 | Calabria_ISS_1011_2017 | Italy | 2017_08_02 | Human |
| MK120200 | Calabria_ISS_1028_2017 | Italy | 2017_08_09 | Human |
| MK120201 | Emilia_Romagna_ISS_1_2007 | Italy | 2007_08_20 | Human |
| MK120202 | Emilia_Romagna_ISS_2_2007 | Italy | 2007_08_25 | Human |
| MK121891 | AMA290 | Brazil_Amazonia_Manaus | 2015_07_15 | Human |
| MK121892 | AMA291 | Brazil_Amazonia_Manaus | 2015_07_15 | Human |
| MK121893 | AMA292 | Brazil_Amazonia_Manaus | 2015_07_15 | Human |
| MK121894 | AMA293 | Brazil_Amazonia_Manaus | 2016_01_31 | Human |
| MK121895 | AMA74 | Brazil_Amazonia_Manaus | 2017_03_20 | Human |
| MK121896 | AMA346 | Brazil_Roraima_Boa Vista | 2017_03_03 | Human |
| MK121898 | AMA352 | Brazil_Roraima_Boa Vista | 2017_02_22 | Human |
| MK121899 | AMA354 | Brazil_Roraima_Boa Vista | 2017_03_17 | Human |
| MK121900 | AMA362 | Brazil_Roraima_Iracema | 2017_03_17 | Human |
| MK121901 | AMA364 | Brazil_Roraima_Boa Vista | 2017_03_17 | Human |
| MK121902 | AMA366 | Brazil_Roraima_Boa Vista | 2017_03_17 | Human |
| MK121903 | AMA368 | Brazil_Roraima_Boa Vista | 2017_03_15 | Human |
| MK121904 | AMA369 | Brazil_Roraima_Boa Vista | 2017_03_02 | Human |
| MK121906 | AMA379 | Brazil_Roraima_Boa Vista | 2017_02_27 | Human |
| MK121907 | AMA381 | Brazil_Roraima_Boa Vista | 2017_02_27 | Human |
| MK121908 | AMA294 | Brazil_Roraima_Boa Vista | 2014_12_03 | Human |
| MK134712 | AMA295 | Brazil_Roraima | 2014_11_11 | Human |
| MK134713 | PHE_2018_Ex_Sudan | Sudan | 2018_10 | Human |
| MK163628 | RJ94 | Brazil_RJ_RJ | 2016_05_02 | Human |
| MK244636 | RJ111 | Brazil_RJ_Mesquita | 2016_04_05 | Human |
| MK244638 | RJ125 | Brazil_RJ_Sao Goncalo | 2017_03_07 | Human |
| MK244640 | Bio_M16 | Brazil_RJ_Niteroi | 2018_03_27 | Human |
| MK244647 | KA_Blore5310 | India | 2015 | Human |
| MK370030 | MH_Nasik275 | India | 2015 | Human |
| MK370031 | DL2 | India | 2016 | Human |
| MK370032 | MH_Pune33 | India | 2016 | Human |
| MK370033 | CHRF_0071_06_17_2017 | Bangladesh | 2017_06_17 | Human |
| MK468608 | CHRF_0103_06_19_2017 | Bangladesh | 2017_06_19 | Human |
| MK468609 | CHRF_0094_11_22_2017 | Bangladesh | 2017_11_22 | Human |
| MK468610 | CHRF_0099_05_31_2017 | Bangladesh | 2017_05_31 | Human |
| MK468611 | CHRF_0101_06_17_2017 | Bangladesh | 2017_06_17 | Human |
| MK468612 | CHRF_0106_06_24_2017 | Bangladesh | 2017_06_24 | Human |
| MK468613 | CHRF_0108_07_10_2017 | Bangladesh | 2017_07_10 | Human |
| MK468614 | CHRF_0110_07_10_2017 | Bangladesh | 2017_07_10 | Human |
| MK468615 | CHRF_0104 | Bangladesh | 2017_06_19 | Human |
| MK468616 | CHRF_0105_06_21_2017 | Bangladesh | 2017_06_21 | Human |
| MK468617 | CHRF_0107_06_29_2017 | Bangladesh | 2017_06_29 | Human |
| MK468618 | CHRF_0109_07_8_2017 | Bangladesh | 2017_07_08 | Human |
| MK468619 | CHRF_0012_07_11_2017 | Bangladesh | 2017_07_11 | Human |
| MK468620 | CHRF_0111_07_18_2017 | Bangladesh | 2017_07_18 | Human |
| MK468621 | CHRF_0112_08_31_2017 | Bangladesh | 2017_08_31 | Human |
| MK468622 | CHRF_0102_06_17_2017 | Bangladesh | 2017_06_17 | Human |
| MK468626 | CHRF_0113_09_18_2017 | Bangladesh | 2017_09_18 | Human |
| MK468627 | TC37 | Thailand | 2018_06_27 | Human |
| MK468801 | PHECHIKV_1_I | India | 2016_09_02 | Human |
| MK473621 | PHECHIKV_2_I | India | 2016_09_13 | Human |
| MK473622 | PHECHIKV_3_I | India | 2016_09_20 | Human |
| MK473623 | PHECHIKV_4_I | India | 2016_10_23 | Human |
| MK473624 | PHECHIKV_5_I | India | 2016_08_26 | Human |
| MK473625 | PHECHIKV_6_I | India | 2016_10_06 | Human |
| MK473626 | PHECHIKV_7_I | India | 2016_10_23 | Human |
| MK473627 | PHECHIKV_8_I | India | 2016_10_13 | Human |
| MK473628 | PHECHIKV_9_I | India | 2016_10_18 | Human |
| MK473629 | PHECHIKV_10_I | India | 2016 | Human |
| MK473630 | PHECHIKV_11_I | India | 2016_09_11 | Human |
| MK473631 | PHECHIKV_12_I | India | 2016_09_17 | Human |
| MK473632 | PHECHIKV_13_I | India | 2016_10_19 | Human |
| MK473633 | PHECHIKV_14_I | India | 2016 | Human |
| MK473634 | PHECHIKV_1_TC | India | 2016_09_02 | Human |
| MK473635 | PHECHIKV_3_1D2 | India | 2016_09_20 | Human |
| MK473636 | PHECHIKV_3_R | India | 2016_09_20 | Human |
| MK473637 | PHECHIKV_3_TC | India | 2016_09_20 | Human |
| MK473638 | PHECHIKV_4_TC | India | 2016_10_23 | Human |
| MK473639 | PHECHIKV_9_TC | India | 2016_10_18 | Human |
| MK473640 | JHCK280 | India | 2016_09_28 | Human |
| MK518340 | BeAr849404 | Brazil_Maranhao_Codo | 2017_05_16 | Human |
| MK518395 | JHCK300 | India | 2016_09_30 | *Aedes aegypti* |
| MK551552 | JHCK308 | India | 2016_10_03 | Human |
| MK551553 | RC_Diosso_2019 | Republic of the Congo | 2019 | Human |
| MK690206 | NK3302 | Thailand | 2018_11_21 | Human |
| MK848202 | PNRCNG_LNSP_INMI1 | Republic of the Congo | 2019_03_20 | Human |
| MK935343 | PNRCNG_LNSP_INMI2 | Republic of the Congo | 2019_03_05 | Human |
| MK935344 | 197_ex_Thailand_2019 | Finland | 2019_02 | Human |
| MN075149 | 212_ex_Thailand_2019 | Finland | 2019_02 | Human |
| MN075150 | 19CHKYN01LY | China | 2019_05_07 | Human |
| MN402883 | 19CHKYN03ZJF | China | 2019_06_28 | Human |
| MN402884 | 19CHKYN02LLZ | China | 2019_05_08 | Human |
| MN402885 | 19CHKYN04CZT | China | 2019_07_02 | Human |
| MN402886 | 19CHKYN08May | Myanmar | 2019_08_01 | Human |
| MN402887 | 19CHKYN09PYINI | Myanmar | 2019_08_01 | Human |
| MN402888 | 19CHKYN05LHL | China | 2019_07_25 | Human |
| MN402889 | 19CHKYN06HYG | China | 2019_07_26 | Human |
| MN402890 | 19CHKYN07LHX | China | 2019_07_30 | Human |
| MN402891 | 19CHKYN10ZZN | China | 2019_08_02 | Human |
| MN402892 | Thail_2019 | Australia | 2019 | Human |
| MN630017 | UNKNOWN_MP174049 | NA | NA | Human |
| MP174049 | Bol1 | Bolivia | 2015_02_24 | Unknown |
| MT150092 | Bol2 | Bolivia | 2015_03_01 | Human |
| MT150093 | Bol3 | Bolivia | 2015_03_01 | Human |
| MT150094 | Bol4 | Bolivia | 2015_04_26 | Human |
| MT150095 | Bol5 | Bolivia | 2015_05_04 | Human |
| MT150096 | Bol6 | Bolivia | 2015_05_04 | Human |
| MT150097 | Bol7 | Bolivia | 2015_05_04 | Human |
| MT150098 | Bol8 | Bolivia | 2015_05_04 | Human |
| MT150099 | Bol9 | Bolivia | 2015_04_27 | Human |
| MT349960 | H542 | Brazil_Mato Grosso_Sinop | 2018_02_07 | Human |
| XXX | 43P41 | Brazil_Pernambuco_Recife | 2016_05_17 | NA |
| XXX | 97P45 | Brazil_Pernambuco_Recife | 2016_06_01 | NA |
| XXX | 53P51 | Brazil_Pernambuco_Recife | 2016_03_30 | NA |
| XXX | 199P195 | Brazil_Pernambuco_Recife | 2016_07_04 | NA |
| XXX | 315P290 | Brazil_Pernambuco_Recife | 2016_08_16 | NA |
