# Supplementary material for: The Emergence of Chikungunya ECSA Lineage in a Mayaro Endemic Region on the Southern Border of the Amazon Forest
Source: Trop Med Infect Dis. 2020 Jun 26;5(2):105. doi: 10.3390/tropicalmed5020105 (PMC7345197; doi:10.3390/tropicalmed5020105)
Supplement: Supplementary file 1 [file tropicalmed-05-00105-s001.zip › Supplementary table 3 - CHIKV and MAYV distribution throughout the years.docx]

**Table S3. Chikungunya and Mayaro viruses distribution throughout the years.**

| **Virus/year** | **2014** | **2015** | **2016** | **2017** | **2018** | **TOTAL** |  |
| --- | --- | --- | --- | --- | --- | --- | --- |
| **Tested samples** | **92** | **59** | **10** | **147** | **46** | **354** |  |
| **Negative samples** | **62** | **35** | **6** | **131** | **42** | **276** |  |
| **Arbovirus positive samples*** | **30** | **24** | **4** | **16** | **4** | **78** |  |
| MAYV | 13 | 10 | 4 | 6 | 0 | **33** |  |
| CHIK | 0 | 0 | 0 | 0 | 1 | **1** |  |

*Results for ZIKV, DENV, and SLEV, which are discussed elsewhere (Vieira et al. 2019; Kubiszeski et al. 2020; Moraes et al. 2020).

Vieira, C.J.S.P.; Machado, L.C.; Pena, L.J.; de Morais Bronzoni, R.V.; Wallau, G.L. Spread of two Zika virus lineages in Midwest Brazil. *Infect. Genet. Evol.* **2019**, *75*, 103974, <https://doi.org/10.1016/j.meegid.2019.103974>.

Kubiszeski, J.R.; Vieira, C.J.S.P.; Thies, S.F.; Silva, D.J.F.; Barreto, E.S.; Mondini, A.; Bronzoni, R.V.M. Detection of the Asian II genotype of dengue virus serotype 2 in humans and mosquitoes in Brazil. *Rev. Soc. Bras. Med. Trop.* **2020**, 53, e20190439, <http://dx.doi.org/10.1590/0037-8682-0439-2019>.

Moraes, M.M.; Kubiszeski, J.R.; Vieira, C.J.S.P.; Gusmao, A.F.; Pratis, T.S.; Colombo, T.E.; Thies, S.F.; Araujo, F. do C.; Zanelli, C.F.; Milhim, B.H.G. de A.; et al. Concomitant detection of Saint Louis encephalitis virus in two Brazilian States. *Mem. Inst. Oswaldo Cruz* (under review).
